# Investigation of the impact of educational attainment on dementia mortality: A Danish nationwide comparative cohort study

**DOI:** 10.1002/alz.71945

**Published:** 2026-11-05

**Authors:** Janet Janbek, Thomas Munk Laursen, Sandra Feodor Nilsson, Carsten Bøcker Pedersen, Dagny Ros Nicolaisdottir, Ann Nielsen, Henrik Brønnum‐Hansen, Gunhild Waldemar

**Affiliations:** ^1^ Danish Dementia Research Centre, Department of Neurology Copenhagen University Hospital – Rigshospitalet Copenhagen Denmark; ^2^ National Centre for Register‐based Research Aarhus University Aarhus Denmark; ^3^ Mental Health Centre Copenhagen Copenhagen University Hospital – Bispebjerg and Frederiksberg Copenhagen Denmark; ^4^ Hammel Neurorehabilitation Centre and University Research Clinic Aarhus University Hammel Denmark; ^5^ Department of Social Medicine University of Copenhagen Copenhagen Denmark; ^6^ Department of Clinical Medicine University of Copenhagen Copenhagen Denmark

**Keywords:** delay of onset, dementia, education, life expectancy, mortality, survival

## Abstract

**INTRODUCTION:**

We aimed to investigate the impact of education on mortality in individuals with dementia, adding a birth cohort perspective.

**METHODS:**

This was a register‐based cohort study of 65‐ to 100‐year‐olds between 2000 and 2022. Exposure was dementia, outcome was death, and the main stratification variable was education. We estimated all‐cause and cause‐specific mortality rate ratios (MRRs) and overall and cause‐specific life‐years lost (LYL), by sex and birth cohorts.

**RESULTS:**

In 1,991,379 persons, MRRs were higher in dementia, highest in long‐term education, and in more recent birth cohorts. Dementia was associated with reductions of 5.01 (men) and 5.29 years (women) in the long‐term education group, and 3.99 years (men and women) in the short‐term education group. The most pronounced excess mortality and most LYL were for dementia and the nervous system.

**DISCUSSION:**

Findings highlight the burden of dementia and education as a target for delaying its onset and ensuring more dementia‐free years.

## Highlights

- Nationwide study: impact of education across birth cohorts on mortality in dementia.
- Persons with longer‐term education had longer life expectancy in the absence of dementia.
- Dementia shortens a larger portion of remaining life.
- Education is a powerful predictor of survival and is more influential in modern generations.

## 1 BACKGROUND

Recent evidence shows that dementia incidence rates have declined in Europe and the United States over the past 25 years,^1^ which may be due to various lifestyle interventions and policies. However, dementia‐related mortality is increasing in most countries.^2^, ^3^ While significantly enhanced survival rates of acute cardiovascular disease and cancer have been achieved, life expectancy has increased, and dementia has become more prevalent, dominating the burden of disease today. In Denmark, between 2000 and 2025, life expectancy increased from 74.5 years to 80.3 years for men and from 79.2 years to 83.9 years for women.^4^ In a previous Danish nationwide register‐based cohort study based on more than two million individuals, it was shown that up to 10 years of life expectancy were lost in age groups 65+ years with dementia, compared with the general population.^5^ In addition, dementia as the underlying cause of death accounted for the majority of life‐years lost (LYL) among individuals with dementia, recognizing dementia as a life‐threatening condition and highlighting the urgent need for targeted public health and policy responses for dementia prevention.

There is consistent evidence that short‐term education is associated with a higher risk of dementia.^6^, ^7^, ^8^ However, previous results on the associations between educational level and mortality in persons with dementia are inconsistent. Some studies have shown that more highly educated people exhibit poorer survival following dementia,^9^, ^10^, ^11^ a potential reflection of higher cognitive reserve that may postpone symptoms of dementia and such people are diagnosed at more severe stages of underlying brain pathology.^10^ Other studies have shown no association between higher education and poorer survival following dementia.^12^, ^13^, ^14^, ^15^ Evidence on the association between education and mortality in dementia remains inconclusive and previous studies were hindered by generally small sample sizes (often single clinic‐based samples) and inconsistencies in measures across studies, for example, reference groups for mortality estimates.^10^, ^11^

Moreover, with shifts in population demographics and educational attainment over time, risk profiles have also changed.^16^, ^17^ Therefore, the impact of education on mortality in dementia may differ across birth cohorts due to varying underlying concepts reflected by education (e.g., social norms and policies, innate abilities, risk profiles of lifestyle and disease), and differences in baseline risks for dementia and mortality. Variations in all‐cause mortality across birth cohorts have been shown in the general population,^18^ but insights into dementia are lacking. Such knowledge can unveil potential clues of the trajectories of life experiences that help us understand risk factors for dementia.

There is therefore a need to expand our understanding of the impact of education on survival in people with dementia, to provide evidence of social inequality in survival among dementia patients, and thereby to contribute knowledge valuable for targeted dementia prevention and delay of onset efforts. Further, to our knowledge, associations between education and mortality in dementia have not yet been investigated in Denmark.

We aimed to investigate the impact of education on all‐cause and cause‐specific mortality in individuals with dementia as compared with people without dementia and the general population, using nationwide register data and adding a birth cohort perspective.

**RESEARCH IN CONTEXT**

1. **Systematic review**: We reviewed the literature for studies on the impact of education on mortality and survival following dementia diagnosis. There is consistent evidence that short‐term education is associated with a higher risk of dementia. However, previous results on the associations between educational level and mortality in persons with dementia are inconsistent. We aimed to investigate the impact of education on all‐cause and cause‐specific mortality in individuals with dementia using nationwide register data and adding a birth cohort perspective.
2. **Interpretation**: In 1,991,379 individuals (52% women), all‐cause mortality was substantially higher in dementia, with MRRs highest in the long‐term education group and lowest in the short‐term education group. MRRs varied across cause and education. The relative mortality burden associated with dementia was greatest in more recent birth cohorts. Dementia was associated with absolute reductions of 5.01 years for men and 5.29 years for women in the long‐term education group and 3.99 years for men and women in the short‐term education group. The most pronounced excess mortality and most LYL were associated with dementia, followed by the nervous system. While individuals with longer‐term education had longer life expectancy in the absence of dementia, dementia shortens a larger portion of their potential remaining life.
3. **Future directions**: Our findings highlight the burden of dementia and focus on educational attainment as a powerful predictor of survival that becomes even more influential as society progresses and in modern generations.

## 2 METHODS

### 2.1 Setting and data sources

Using the Civil Registration System,^19^ which covers all residents living in Denmark since 1968, we linked population data to the following national registers: the National Patient Register,^20^ the Psychiatric Central Research Register,^21^ the National Prescription Registry,^22^ the Register of Causes of Death,^23^ and Education Registers.^24^

### 2.2 Study design and population

This is a register‐based cohort study, including all individuals aged 65 to 100 years between January 1, 2000, and December 31, 2022. The cohort consisted of people born and residing in Denmark at the start of follow‐up, defined as age 65 or the study's start date (whichever came later). Exclusion criteria were (a) persons with a prior dementia diagnosis (defined below), (b) persons born before 1920, in accordance with the recommendation outlined in a recent paper to ensure completeness of education data,^25^ and (c) persons with missing education information. Thus, birth cohorts from 1920 to 1957 were included. Individuals were followed up until their death, emigration, or the study's end on December 31, 2022.

We included information on sex, age, calendar period, dementia, death, and education, as described in the following.

### 2.3 Exposure: dementia

Dementia was defined as the first recorded diagnosis in the secondary hospital system in the National Patient Register (International Classification of Diseases, 10th Revision codes) or the first time a prescription for dementia medications was redeemed (Anatomical Therapeutic Chemical codes); see Appendix Table A1 for dementia codes.

### 2.4 Stratification variable: education

The highest attained educational level was extracted from the Education Registers and defined following a recent publication in Denmark, aligned with the International Standard Classification of Education.^25^ The registers are longitudinal and annually record the highest completed education for each individual based on records delivered by educational institutions. Three categories for education were defined: short‐term, intermediate‐term, and long‐term (see Appendix Table A2 for the definition). The highest attained educational level at age 65 was included and treated as a time‐fixed variable.

### 2.5 Outcome: death

We used the Register of Causes of Death for the date and cause of death,^23^ detailed elsewhere.^5^ The coding program IRIS, an automatic system for coding multiple causes of death and for the selection of the underlying cause of death, was used from 2010 (before 2010 ACME). We regrouped causes of death into 13 non‐overlapping causes (Appendix Tables A3 to A5): cancer, diabetes/anemia, other mental disorder (excluding dementia), nervous system (excluding dementia), heart, cerebrovascular, artery/veins, respiratory, gastrointestinal, ill‐defined symptoms, external, and dementia. The remaining causes that are not elsewhere listed, with few deaths, were also included in a separate category as “Other.”

### 2.6 Data analysis

#### 2.6.1 All‐cause and cause‐specific mortality rate ratios (MRRs)

We estimated MRRs and 95% confidence intervals (CIs) comparing individuals with dementia to those without dementia. We used Poisson regression with person‐years at risk as an offset variable. This approximates a Cox regression under the assumption of piecewise proportional rates in age and calendar periods.^26^, ^27^ MRRs were adjusted for age and calendar year (2‐year intervals), resulting in age‐ and calendar‐specific rates. Dementia was modeled as a time‐dependent variable, ensuring that individuals only contributed exposed person‐time after diagnosis and thereby avoid any immortal time bias. All MRRs were stratified by sex, education (short‐term, intermediate‐term, long‐term), and for all‐cause mortality, additionally by birth cohort (1920 to 1929, 1930 to 1939, 1940 to 1957). We applied a similar method for each of the causes of death and stratified by sex and education. MRRs were calculated using SAS 9.4.

#### 2.6.2 Overall and cause‐specific life expectancy and LYL

Life expectancies were calculated for persons with dementia and compared with the general population. Here, we used the general population as the reference group instead of people without dementia. This is a standard method so that life expectancy estimates for the general population are comparable to official national numbers. We calculated the average remaining years of life at each age (65 to 99 years) by integrating the area under and over the survival curve, relying on the mortality rates of people with dementia (age‐specific). We then subtracted the life expectancies at each age from the age of 100 years to calculate the average LYL. Age‐specific estimates were averaged by weighting by the observed age at onset distribution of dementia. Refer to our previous publication for the age at onset distribution.^5^ The same was done for the general population at ages corresponding to the dementia distribution. Finally, the difference between LYL in dementia and the general population was calculated.

For the cause‐specific estimations, we followed the same method for each of the 13 specific causes of death using a decomposition model based on a competing‐risk framework. Using non‐parametric bootstrapping with 1000 resamples of the dementia population, we estimated 95% CIs for the LYL. We assumed no sampling variation for the general population as data were provided from nationwide registers for the entire population. LYL were calculated using R (lillies package).^28^ LYL were stratified by sex and education.

#### 2.6.3 Sensitivity analyses

We conducted two sensitivity analyses. First, we estimated the partial life expectancies (age group 65 to 80) in those with dementia and the general population to further explore the impact of social inequality. In the second analysis, we stratified the population by age groups (65 to 79 years, 80 to 89 years, and 90+ years) to complement our birth cohort analyses and assess whether there was a differential impact of education on mortality.

## 3 RESULTS

The study cohort included all individuals born in Denmark between 1920 and 1957. Of the 2,094,701 eligible individuals, 103,322 (4.9%) had missing information on educational attainment and were excluded, leaving an analytic sample of 1,991,379 persons (52% women). During follow‐up, 794,106 individuals died, corresponding to a cumulative mortality proportion of 39.9%. The analytic cohort comprised 364,673 individuals with long‐term, 816,280 with intermediate‐term, and 810,426 with short‐term education (Table 1). During follow‐up, 158,376 individuals (8%) were diagnosed with dementia.

**TABLE 1:** Study population characteristics.

Characteristic	*N*	Percentage (%)	*N* deaths	Percentage (%)
**Total individuals**	1,991,379	100	794,106	100
**Women**	1,033,948	52	390,061	49
**Men**	957,431	48	404,045	51
**Education**				
Short‐term	810,426	41	431,651	54
Intermediate‐term	816,280	41	273,671	35
Long‐term	364,673	18	88,784	11
**Dementia**	158,376	8	115,310	15

### 3.1 All‐cause mortality rate ratios

All‐cause mortality was substantially higher among individuals with dementia (Figure 1), with MRRs highest in the long‐term education group (women 4.21 [95% CI 4.09, 4.32]; men 4.03 [3.93, 4.13]) and lowest in the short‐term education group (women 2.92 [2.89, 2.95]; men 3.02 [2.97, 3.06]). This was also seen within each birth cohort (Figure 1).

**FIGURE 1:**
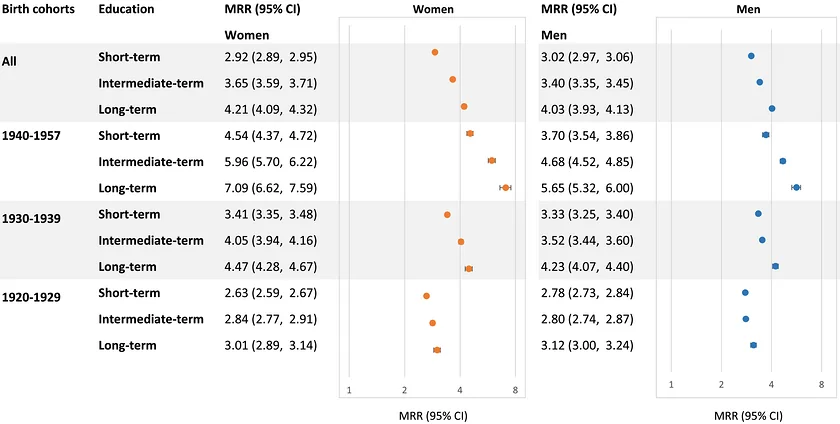
All‐cause MRRs by sex, educational levels, and birth cohorts (in dementia, reference: no dementia). Reference category for all MRRs is *individuals without dementia* within the same sex and educational group. MRRs were adjusted for age and calendar year. CI, Confidence interval; MRR, mortality rate ratio.

A clear cohort gradient was also evident: The youngest cohort (born 1940 to 1957) showed the largest relative risk of death associated with dementia, while the oldest cohort (born 1920 to 1929) showed the smallest. This pattern was consistent across sexes and education levels, although to varying degrees. For example, MRRs among women with long‐term education declined from 7.09 (6.62, 7.59) in the 1940 to 1957 cohort to 3.01 (2.89, 3.14) in the 1920 to 1929 cohort, with similar declines observed among men (from 5.65 [5.32, 6.00] in the 1940 to 1957 cohort to 3.12 [3.00, 3.24] in the 1920 to 1929 cohort). Among those with a low level of education, the decline was smaller, resulting in a reduced difference between the education groups (Figure 1).

### 3.2 Cause‐specific mortality rate ratios

Across all educational groups, dementia was associated with substantially elevated cause‐specific mortality among both women and men (Table 2). MRRs were significantly elevated for every cause of death examined. The magnitude of MRRs varied across cause and education. For most causes – including other mental disorders, nervous system diseases, cerebrovascular diseases, diabetes, and respiratory diseases – MRRs increased with higher educational attainment, with the largest relative risks observed among individuals with long‐term education. After dementia as the cause of death, the highest MRRs were for the nervous system (e.g., women MRR in the long‐term education group 9.84) and other mental disorders (e.g., women MRR in the long‐term education group 5.21). Dementia‐related mortality showed the strongest educational gradients, with the highest MRRs in the long‐term education groups for both sexes (women MRR 55.74 and men MRR 77.44 vs MRRs in short‐term education: women MRR 34.10 and men MRR 59.80). For external causes, the gradient was less pronounced and did not follow the same monotonic pattern observed for most other causes. See Appendix Table A6 and Figure A1 for the number and distribution of causes of death in this cohort.

**TABLE 2 alz71945-tbl-0002:** Cause‐specific MRR by sex and educational level (in dementia, reference: no dementia)

	Women MRR (95% CI)			Men MRR (95% CI)		
Cause of death	Short‐term education	Intermediate‐term education	Long‐term education	Short‐term education	Intermediate‐term education	Long‐term education
**Overall**	2.92 (2.89, 2.95)	3.65 (3.59, 3.71)	4.21 (4.09, 4.32)	3.02 (2.97, 3.06)	3.40 (3.35, 3.45)	4.03 (3.93, 4.13)
**Dementia**	34.10 (33.01, 35.22)	50.47 (47.97, 53.10)	55.74 (51.30, 60.56)	59.80 (56.74, 63.03)	78.70 (74.38, 83.26)	77.44 (70.80, 84.69)
**Cancer**	1.08 (1.04, 1.12)	1.19 (1.13, 1.25)	1.34 (1.23, 1.47)	1.13 (1.08, 1.17)	1.20 (1.15, 1.25)	1.37 (1.27, 1.47)
**Diabetes**	2.34 (2.22, 2.48)	3.14 (2.86, 3.46)	3.40 (2.86, 4.03)	2.82 (2.62, 3.03)	3.34 (3.09, 3.62)	3.54 (3.05, 4.11)
**Other mental disorders**	3.75 (3.26, 4.31)	4.34 (3.52, 5.35)	5.21 (3.80, 7.15)	4.47 (3.85, 5.20)	5.71 (4.90, 6.65)	6.62 (5.04, 8.69)
**Nervous system**	4.76 (4.43, 5.12)	7.19 (6.53, 7.91)	9.84 (8.60, 11.26)	9.25 (8.58, 9.98)	12.85 (11.98, 13.78)	14.15 (12.74, 15.71)
**Heart disease**	1.83 (1.77, 1.88)	2.07 (1.96, 2.18)	2.47 (2.27, 2.69)	2.01 (1.94, 2.09)	2.04 (1.96, 2.13)	2.44 (2.27, 2.63)
**Cerebrovascular**	3.15 (3.03, 3.26)	3.76 (3.54, 3.99)	4.07 (3.70, 4.49)	4.21 (4.01, 4.42)	4.52 (4.28, 4.76)	5.13 (4.69, 5.61)
**Arterial disease**	1.75 (1.61, 1.91)	2.08 (1.81, 2.39)	2.45 (1.93, 3.09)	1.93 (1.74, 2.15)	2.09 (1.87, 2.33)	2.33 (1.91, 2.83)
**Respiratory**	1.97 (1.90, 2.03)	2.52 (2.39, 2.65)	3.03 (2.76, 3.32)	2.42 (2.33, 2.52)	2.67 (2.56, 2.79)	3.43 (3.18, 3.72)
**Gastrointestinal**	1.89 (1.77, 2.01)	2.32 (2.11, 2.57)	2.63 (2.21, 3.12)	2.60 (2.39, 2.83)	2.74 (2.51, 3.00)	3.49 (2.99, 4.08)
**Ill‐defined**	2.32 (2.19, 2.46)	2.87 (2.62, 3.14)	3.72 (3.24, 4.28)	2.15 (1.98, 2.34)	2.90 (2.66, 3.16)	3.24 (2.82, 3.72)
**External causes**	2.96 (2.76, 3.17)	3.21 (2.88, 3.58)	2.66 (2.21, 3.20)	2.79 (2.55, 3.06)	3.40 (3.10, 3.74)	3.34 (2.85, 3.92)
**Other**	1.95 (1.87, 2.03)	2.38 (2.23, 2.53)	2.49 (2.25, 2.77)	2.20 (2.07, 2.33)	2.48 (2.34, 2.64)	2.32 (2.10, 2.56)

*Note*: Reference category for all MRRs is *individuals without dementia* within the same sex and educational group.

Abbreviations: CI, confidence interval; MRR, mortality rate ratio.

### 3.3 Overall life expectancy and LYL

Table 3 presents the overall life expectancy weighted by dementia‐specific mortality. Life expectancy and LYL varied systematically by sex, educational attainment, and dementia status. In both sexes, higher education was associated with longer life expectancy, and women consistently outlived men across all educational strata. Individuals with dementia had markedly shorter life expectancy than the general population, and the difference was largest in the long‐term education group. The proportion of expected years in dementia (out of the total population) tends to decrease with educational level for both sexes: 53.5%, 51.0%, and 50.2% for women with short‐term, intermediate‐term, and long‐term education; and 48.5%, 48.2%, and 46.8% for men with short‐term, intermediate‐term, and long‐term education (percentages based on life[Table alz71945-tbl-0002] expectancies in Table 3). Among men, dementia was associated with absolute reductions of 5.01 years in life expectancy (4.94, 5.09) in the long‐term education group, 4.50 years (4.46, 4.55) in the intermediate‐term‐education group, and 3.99 years (3.95, 4.03) in the short‐term education group. Corresponding reductions among women were 5.29 years (5.20, 5.38), 4.84 years (4.78, 4.89), and 3.99 years (3.96, 4.02), respectively.

**TABLE 3:** Overall life expectancy weighted by dementia‐specific mortality, by sex and education level.

Sex	Education	Status	Life expectancy
Women	**Short‐term**	Total population	8.58
		Individuals with dementia	4.59
		**Difference (life years lost)**	**3.99 (3.96, 4.02)** ^a^
	**Intermediate‐term**	Total population	9.87
		Individuals with dementia	5.03
		**Difference (life years lost)**	**4.84 (4.78, 4.89)** ^a^
	**Long‐term**	Total population	10.63
		Individuals with dementia	5.34
		**Difference (life years lost)**	**5.29 (5.20, 5.38)** ^a^
Men	**Short‐term**	Total population	7.75
		Individuals with dementia	3.76
		**Difference (life years lost)**	**3.99 (3.95, 4.03)** ^a^
	**Intermediate‐term**	Total population	8.71
		Individuals with dementia	4.20
		**Difference (life years lost)**	**4.50 (4.46, 4.55)** ^a^
	**Long‐term**	Total population	9.42
		Individuals with dementia	4.41
		**Difference (life years lost)**	**5.01 (4.94, 5.09)** ^a^

*Note*: The table reports remaining life expectancy from age 65, calculated using a weighted average across ages 65 to 99.

^a^ Bootstrap estimation of confidence intervals based on 1000 iterations. Minor discrepancies (≤0.01 years) in “Difference” reflect rounding.

### 3.4 Cause‐specific LYL

Cause‐specific patterns in LYL showed that individuals with dementia generally experienced greater losses than the general population for most causes of death across all educational groups (Figure 2, Appendix Tables A7 to A8). The magnitude of these losses varied: Men and women with longer‐term education tended to experience more pronounced negative LYL for several causes, particularly nervous system diseases, whereas those with short‐term education showed somewhat smaller deficits. A small but consistent exception is cerebrovascular disease, where men and women with short‐term education lost marginally more life‐years than those with longer‐term education. Cancer and heart disease consistently showed positive LYL values, indicating fewer LYL to these causes. These positive LYL estimates were relatively stable across education levels, with only modest variation.

**FIGURE 2:**
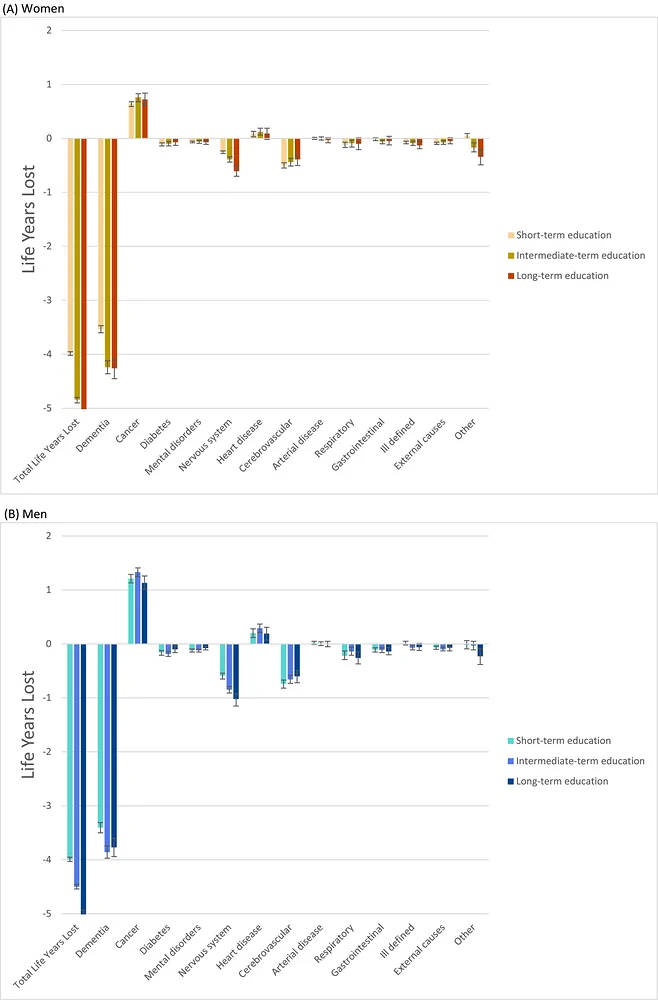
Life years lost by cause of death in (A) women and (B) men, and by educational levels.

### 3.5 Sensitivity analyses

Considering the 65 to 80 age range, educational differences in LYL were markedly attenuated (Table A9, Figure A2). In contrast, when LYL were calculated over the full remaining lifespan, the expected educational gradient re‐emerged. In the second sensitivity analysis stratified by underlying age group, MRRs were higher in the group with long‐term education across all age groups (65 to 80, 81 to 90, and 91+ years), but the difference between the groups with long and short‐term education was largest in the 65 to 80 years group (Table A10).

## 4 DISCUSSION

In this nationwide Danish cohort study, we assessed the impact of educational attainment across birth cohorts on mortality among individuals with dementia. We found that all‐cause mortality was higher in dementia (vs no dementia), and people with dementia had shorter life expectancy than the general population, with higher excess mortality and greater reductions in life expectancy in individuals with longer‐term education. The relative mortality burden associated with dementia was greatest in more recent birth cohorts, and the education gradient was similar across all birth cohorts (higher mortality in individuals with longer‐term education). Similar patterns were observed in women and men. Women and men exhibited broadly similar cause‐specific death profiles, with the most pronounced excess mortality and most LYL for dementia, followed by nervous system, and the steepest educational gradients for these categories, where highly educated individuals with dementia experienced the greatest additional losses and highest excess mortality.

### 4.1 Interpretation and previous literature

Several insights can explain our findings. In the absence of dementia, the baseline rate of mortality is lower in individuals with longer‐term education, probably owing to a generally healthier lifestyle, better living conditions, and overall health reflected in decreased disease burden.^29^ Therefore, the impact of dementia will be much greater in individuals with longer‐term education (higher MRRs in the current study). Further, the onset of dementia shortens a larger portion of the potential remaining life, leading to a greater absolute number of LYL among highly educated individuals.

We provide potential explanations of our findings in the following. However, we note that additional information that we did not have on, for example, dementia severity, time from symptom onset to diagnosis, and cognitive trajectories would be crucial to move our explanations from speculative to evidence based. It is possible that individuals with dementia and longer‐term education are diagnosed with dementia at later stages and more advanced brain pathology and so have shorter life expectancies following diagnosis. Here, we can draw on the cognitive reserve hypothesis. It has been proposed that longer‐term education and, therefore, greater cognitive reserve will lead to the ability to experience more brain pathology before presenting with dementia symptoms.^10^ This is supported by previous findings that Alzheimer's disease‐related pathologic changes in the brain were more advanced among subjects with dementia who had a high level of educational attainment than in those with a low level of education, given similar clinical dementia severity.^10^, ^30^ Other studies have shown that cognitive decline accelerates after a dementia diagnosis in individuals with longer‐term education.^31^ However, findings from observational studies on whether longer‐term education is associated with shorter survival in dementia are generally inconsistent, likely influenced by varying settings and related system‐level and societal factors.^9^, ^10^, ^11^, ^12^, ^13^, ^14^, ^15^ As most of the previous studies were based on small sample sizes and lacked nationwide coverage, our findings from the current study, based on nationwide data, provide an important contribution. If individuals with longer‐term education are diagnosed with dementia at later stages of pathology, they experience more years lived before showing dementia symptoms, which can mean that targeting education can delay the onset of dementia symptoms. This is crucial knowledge, as it has been shown based on UK data that delaying the onset of disease by 5 years may result in a 30% reduction of dementia incidence.^32^

On the other hand, potential ascertainment bias can mean that people with longer‐term education are diagnosed with dementia at earlier rather than later stages of pathology. For example, people who are highly educated may have better access to healthcare and increased awareness of dementia, so they may be diagnosed with dementia at earlier stages. However, if this is true, people with longer‐term education should have longer life expectancy if they are diagnosed with dementia earlier, but our data showed the opposite for life expectancy.

It is important to note that educational attainment can reflect several constructs, and disentangling these will inform targeted interventions. Longer‐term education can reflect innate abilities, social norms/environment that define the opportunities to attain education, and the consequences of not attaining education that shape one's life trajectories, health literacy, access to healthcare, and burden of diseases. Our analyses considering the birth cohort effect provide interesting insights in this regard. We observed that the youngest birth cohorts (born 1940 to 1957) with dementia showed the greatest relative risk of all‐cause death. Similar observations were reported in the general population (without focus on dementia) in a previous study with data from 14 European countries.^18^ As such, education is not just a proxy of innate abilities, but a critical marker of the social and environmental shifts that define a person's life trajectory. In older cohorts, having little education was not uncommon, and it did not necessarily signal extreme disadvantage. In younger cohorts, less education, particularly in a society where it is accessible, can reflect underlying disadvantages such as health issues, lack of social support, or poverty. Consequently, the gap between those with more and less education becomes more polarized in modern generations, particularly in high‐income countries.^18^ Moreover, the consequences of not attaining higher levels of education may be even more detrimental to one's life expectancy in younger cohorts, including through the lack of the cognitive reserve that may help delay dementia.

Our findings on cause‐specific mortality highlighted the burden of dementia as a cause of death, as well as other mental disorders and diseases of the nervous system. These were the causes with the largest excess mortality and the most pronounced negative LYL across all educational groups and were greatest in individuals with long‐term education. It is also possible that because of the high burden of comorbidity in people with dementia and short‐term education, the registration of dementia as a cause of death is less likely, as other comorbidities are prioritized. Noteworthy exceptions among the causes of death were cancer and heart disease, where fewer life‐years were lost to these causes. We noted this exception in our previous study,^5^ likely explained by nihilistic attitudes or a lack of actively capturing and treating these diseases in persons with dementia. In the current study, we also note the minor variation in LYL across education groups for cancer and heart diseases, which is different than in other causes of death. This further suggests that the LYL observed do not reflect true disease burden but rather the lack of diagnosis of these diseases in people with dementia. Moreover, future efforts should explore in detail other mental disorders and external causes to explore and explain what these findings are attributed to.

Finally, our results show that level of education has a stronger impact on mortality in the 65‐ to 80‐year age group. In older ages, the difference between educational levels is small, likely because other biological mechanisms become the main drivers of mortality in older age. In addition, survivor bias should be noted in this context. People with short‐term education who live to older ages may be seen as “survivors” who did not die of diseases. Therefore, at older ages, survivors with short‐term education and those with long‐term education become more similar biologically, as reflected in the slight differences in MRRs in older ages between the education groups.

### 4.2 Methodological considerations

Our study provides notable strengths over available evidence. We utilized nationwide data to comprehensively assess the impact of education on life expectancy in dementia, focusing on both all‐cause and cause‐specific mortality, quantifying LYL following a dementia diagnosis, comparing the impact of education on mortality and life expectancy in individuals with dementia versus those without dementia and the general population, and assessing the birth cohort trends in mortality. Some limitations should also be noted. First, we did not have data on ethnicity, which may limit external generalizability. However, only 9% of the population in Denmark in 2022 were non‐Western immigrants or their descendants.^33^ Future work should replicate our findings while considering dementia severity to confirm that persons with longer‐term education are diagnosed at later stages of the pathology. In addition, future work should replicate our approach to compare educational impact from different healthcare and social systems, identifying system‐level variations important for dementia risk reduction measures. Second, the Register of Causes of Death has unknown validity (and the likelihood of receiving the correct cause of death may vary among individuals in older populations), and this should be taken into consideration when interpreting our findings on cause‐specific mortality and LYL. Further, while selection bias was limited because data on education were obtained from registries rather than self‐reports, potential bias due to missing data cannot be ruled out. Finally, we included sex, age, and calendar period as confounders in our analyses in order to show life expectancy and MRR irrespective of other potential covariates. However, any factors that systematically vary across educational groups and predict mortality would cause residual confounding. This includes, for example, variations in healthcare access and seeking behavior that is linked to the level of education, which can determine getting a dementia diagnosis and predict other comorbidities and overall and cause‐specific survival. In addition, the younger cohorts in our study were observed at younger ages and may differ systematically from older cohorts in terms of their comorbidity profiles and overall and cause‐specific mortality; therefore, the birth cohort effects must be interpreted considering this.

### 4.3 Conclusions and implications

While individuals with longer‐term education had longer life expectancy in the absence of dementia, dementia shortens a larger portion of their potential remaining life. Our findings highlight the burden of dementia and put educational attainment in focus as a powerful predictor of survival that becomes even more influential as society progresses and in modern generations.

## CONFLICT OF INTEREST STATEMENT

Janet Janbek reports consultancy work for Eli Lilly. The remaining authors have no conflicts of interest to report. Author disclosures are available in the Supporting Information.

## CONSENT STATEMENT

This study is based on danish national registry data, and Danish law does not require ethical approval or consent.

## Supporting information




Supporting Information

Supporting Information
