# Supplementary material for: Investigation of the impact of educational attainment on dementia mortality: A Danish nationwide comparative cohort study
Source: Alzheimers Dement. 2026 Nov 5;22(11):e71945. doi: 10.1002/alz.71945 (PMC13740376; doi:10.1002/alz.71945)
Supplement: Supplementary file 2 [file ALZ-22-e71945-s001.docx]

**Appendix – Change of the impact of educational attainment on the burden of dementia: a Danish nationwide comparative cohort study**

Authors: Janet Janbek, Thomas Munk Laursen, Sandra Feodor Nilsson, Carsten Bøcker Pedersen, Dagny Ros Nicolaisdottir, Ann Nielsen, Henrik Brønnum-Hansen, Gunhild Waldemar

Contents

[Table A1: Definition of dementia	2](#_Toc227583668)

[Table A2. Education definition	3](#_Toc227583669)

[Table A3: Definition of causes of death	3](#_Toc227583670)

[Table A4: Causes of death - “List 49” English and Danish description.	5](#_Toc227583671)

[Table A5: Causes of death – “List 49” ICD-8 and ICD-10 definition.	7](#_Toc227583672)

[Table A6. Number and distribution of causes of death	8](#_Toc227583673)

[Figure A1. Distribution of causes of death	8](#_Toc227583674)

[Table A7. Life Years Lost (LYL; ages 65–99) among women with dementia relative to the general population, stratified by cause of death	9](#_Toc227583675)

[Table A8. Life Years Lost (LYL; ages 65–99) among men with dementia relative to the general population, stratified by cause of death	10](#_Toc227583676)

[Table A9. Educational differences in life years lost under alternative upper age limits (80 vs. 100 years)	11](#_Toc227583677)

[Figure A2. Educational differences in life years lost under alternative upper age limits (80 vs. 100 years)	11](#_Toc227583678)

[Table A10. Mortality Rate Ratios in women and men across education groups, stratified by the underlying age.	12](#_Toc227583679)

## Table A1: Definition of dementia

| Dementia type | ICD8 | ICD10 |
| --- | --- | --- |
| Alzheimer’s disease | 290.10 | F00.*, G30.* |
| Vascular dementia | 293.09-19 | F01.* |
| Frontotemporal dementia | 290.11 | F02.0*, G31.0A, G31.0B |
| Dementia without specification | 290.09, 290.19 | F03.*, G31.9 |
| Other dementias | 290.18 | G31.8, G31.8E, F02.1-F02.8 |

To ensure that no individuals had a dementia diagnosis in the Danish Patient Register prior to 1994, when ICD-8 was used in Denmark, we will exclude those with ICD-8 dementia codes

| Dementia medication | ATC code |
| --- | --- |
| Cholinesterase inhibitors |  |
| Donepezil | N06DA02 |
| Rivastigmine | N06DA03 |
| Galantamine | N06DA04 |
| Glutamate-receptor antagonists memantine | N06DX01 |

## Table A2. Education definition

Table from: Hjorth, C., Kjærulff, T., Thomsen, M., Cronin-Fenton, D., Dalton, S., & Olsen, M. (2025). SEPLINE: Socioeconomic Position in Epidemiological Research—A National Guideline on Danish Registry Data. *Clinical Epidemiology*, *Volume 17*, 593–624. <https://doi.org/10.2147/CLEP.S520772>. We refer to “Short education”, “Medium education”, and “Long education” in our manuscript as “Short-term education”, “Intermediate-term education”, and “Long-term education”, respectively.

## Table A3: Definition of causes of death

| Disease category | 49 list |
| --- | --- |
| Other/not elsewhere classified | 01, 02, 15, 36, 37, 38, 39, 40, 41, 42, 43, 44 |
| Cancer | 03, 04, 05, 06, 07, 08, 09, 10, 11, 12, 13, 14 |
| Diabetes/blood | 16, 17, 18 |
| Mental | 19 (excluding dementia codes) |
| Nervous system | 20, 21, 22 (excluding dementia codes) |
| Heart | 23, 24, 25 |
| Cerebrovascular | 26 |
| Artery | 27, 28 |
| Respiratory | 29, 30, 31, 32, 50 (Covid etc.) |
| Gastrointestinal | 33, 34, 35 |
| Ill-defined symptoms | 45 |
| External (unnatural) | 46, 47, 48, 49 |
| Dementia | Not in 49-list, incl. dementia codes from Mental/Nervous system |

## Table A4: Causes of death - “List 49” English and Danish description.

|  | English description | Danish description |
| --- | --- | --- |
| 1 | Infectious diseases (excl. tuberculosis) | Infektionssygdomme (excl. tuberkulose) |
| 2 | Tuberculosis | Tuberkulose |
| 3 | Cancer of the oral cavity and esophagus | Kræft i mundhule og spiserør |
| 4 | Stomach cancer | Kræft i mavesæk |
| 5 | Intestinal cancer, excluding rectum | Kræft i tarme, undtagen endetarm |
| 6 | Rectal cancer | Kræft i endetarm |
| 7 | Cancer of the larynx, trachea, bronchi, lungs | Kræft i strubehoved, luftrør, bronkier, lunger |
| 8 | Cancer of bones and skin | Kræft i knogler og hud |
| 9 | Breast cancer | Kræft i brystkirtel |
| 10 | Cervical cancer | Kræft i livmoderhals |
| 11 | Cancer of other parts of the uterus | Kræft i andre dele af livmoder |
| 12 | Prostate cancer | Kræft i blærehalskirtel |
| 13 | Cancer in other and unspecified locations | Kræft i andre og uspecificerede lokaliteter |
| 14 | Cancer of lymphatic and hematopoietic tissue | Kræft i lymfatiske og bloddannende væv |
| 15 | Benign tumor and tumor of unspecified nature | Godartet svulst og svulst af uspecificeret natur |
| 16 | Endocrine disorders and deficiency diseases | Endokrine lidelser og mangelsygdomme |
| 17 | Diabetes | Sukkersyge |
| 18 | Diseases of the blood and blood-forming organs | Sygdomme i blod og bloddannende organer |
| 19 | Mental disorders | Mentale lidelser |
| 20 | Meningitis | Hjernehindebetændelse |
| 21 | Diseases of the nervous system and sensory organs | Sygdomme i nervesystem og sanseorganer |
| 22 | Rheumatic fever and its sequelae | Gigtfeber og følgetilstande heraf |
| 23 | Hypertension | Blodtryksforhøjelse |
| 24 | Ischemic heart diseases | Iskæmiske hjertesygdomme |
| 25 | Other heart diseases | Andre hjertesygdomme |
| 26 | Cerebrovascular diseases | Karsygdomme i hjerne |
| 27 | Diseases of arteries, arterioles, and capillaries | Sygdomme i arterier, arterioler og kapillærer |
| 28 | Other diseases of the circulatory system | Andre sygdomme i kredsløbets organer |
| 29 | Acute respiratory infections incl. influenza | Akutte luftvejsinfektioner incl. influenza |
| 30 | Pneumonia | Lungebetændelse |
| 31 | Bronchitis, emphysema, and asthma | Bronkitis, udvidede lunger og astma |
| 32 | Other diseases of the respiratory system | Andre sygdomme i åndedrætsorganer |
| 33 | Diseases of the oral cavity, esophagus, and stomach | Sygdomme i mundhule, spiserør og mavesæk |
| 34 | Diseases of the liver and bile ducts | Sygdomme i lever og galdeveje |
| 35 | Other diseases of the digestive system | Andre sygdomme i fordøjelsesorganer |
| 36 | Nephritis and stones in the urinary organs, etc. | Nyrebetændelse og sten i urinorganer m.m. |
| 37 | Infectious kidney diseases | Infektiøse nyresygdomme |
| 38 | Other diseases of the urinary and genital organs and breast | Andre sygd. i urin- og kønsorg. samt brystkirtel |
| 39 | Pregnancy and childbirth complications in the mother | Svangerskabs- og fødselskompl. hos moderen |
| 40 | Diseases of the skin, bones, musc. system, connective tissue | Sygd. i hud, knogler, bevæg. Syst., bindevæv |
| 41 | Congenital malfor. of the heart and circulatory system | Medfødte misdan. i hjerte og kredsløbsorganer |
| 42 | Other congenital malformations | Andre medfødte misdannelser |
| 43 | Diseases originating in the perinatal period | Sygdomme opstået i perinatalperioden |
| 44 | Senility without mention of psychosis | Senilitet uden oplysning om sindssygdom |
| 45 | Symptoms and ill-defined conditions | Symptomer og mangelfuldt def. tilstande |
| 46 | Traffic accidents | Færdselsulykker |
| 47 | Other accidents | Andre ulykker |
| 48 | Suicide | Selvmord |
| 49 | Other external causes | Andre ydre årsager |
| 50 | Covid, etc. | Covid mm. |

## Table A5: Causes of death – “List 49” ICD-8 and ICD-10 definition.

| List49 | Cause of death, icd-8 and icd-10 codes |
| --- | --- |
| 1 | 000-009, 020-136, A00-A09, A20-A99, B00-B89, B91-B97, B99 |
| 2 | 010-019, A15-A19, B90 |
| 3 | 140-150, C00-C15 |
| 4 | 151 C16 |
| 5 | 152, 153 C17-C19 |
| 6 | 154, C20-C21 |
| 7 | 161, 162 C32-C34 |
| 8 | 170, 172, 173 C40-C44 |
| 9 | 174, C50 |
| 10 | 180, C53 |
| 11 | 181, 182 C54-C55 |
| 12 | 185, C61 |
| 13 | 140-199, C22-C31, C37-C39, C45-C49, C51-C52, C56-C60, C62-C80,C97, D00-D09 |
| 14 | 200-209, C81-C96 |
| 15 | 210-239, D10-D48 |
| 16 | 240-246, 251-279, E00-E07, E15-E90 |
| 17 | 250, E10-E14 |
| 18 | 280-289, D50-D89 |
| 19 | 290-315, F00-F99 |
| 20 | 320, G00-G03 |
| 21 | 321-389, G04-G99, H00-H95 |
| 22 | 390-398, I00-I09 |
| 23 | 400-404, I10-I15 |
| 24 | 410-414, I20-I25 |
| 25 | 420-429, I27, I30-I52 |
| 26 | 430-438, I60-I69 |
| 27 | 440-448, I28, I70-I79 |
| 28 | 450-458, I26, I80-I99 |
| 29 | 460-474, J00-J06, J10-J11 |
| 30 | 480-486, J12-J18 |
| 31 | 490-493, J20-J22, J40-J47 |
| 32 | 500-519, J30-J39, J60-J99 |
| 33 | 520-525, 531-533, 535, K00-K31 |
| 34 | 571, 574, 575, K70-K83 |
| 35 | 520, 577, K35-K67, K85-K93 |
| 36 | 580-584, N00-N08, N20-N23 |
| 37 | 590, 592, 594, N10-N16 |
| 38 | 580-629, N17-N19, N25-N99 |
| 39 | 630-678, O00-O99 |
| 40 | 680-738, L00-L99, M00-M99 |
| 41 | 746, 747, Q20-Q28 |
| 42 | 740-745, 748-759, Q00-Q18, Q30-Q99 |
| 43 | 760-779, P00-P96 |
| 44 | 794, R54 |
| 45 | 780-793, 795, 796, R00-R53, R55-R99 |
| 46 | E 810-E 823, V01-V89 |
| 47 | E 800-807, E825-949, V90-V99, W00-X59, Y40-Y89 |
| 48 | E 950-E 959, X60-X84 |
| 49 | E 960- E999, X85-Y36 |
| 50 | U07.1 R990 (Covid-19) |

## Table A6. Number and distribution of causes of death

| Cause number | Cause name | # deaths | Percent of all deaths |
| --- | --- | --- | --- |
| Cause 1: | Other | 58107 | 7.3 |
| Cause 2: | Cancer | 239842 | 30.2 |
| Cause 3: | Diabetes/blood | 29071 | 3.7 |
| Cause 4: | Mental | 7445 | 0.9 |
| Cause 5: | Nervous | 17297 | 2.2 |
| Cause 6: | Heart | 131294 | 16.5 |
| Cause 7: | Cerebrovascular | 55405 | 7.0 |
| Cause 8: | Artery | 19818 | 2.5 |
| Cause 9: | Respiratory | 104266 | 13.1 |
| Cause 10: | Gastrointestinal | 30666 | 3.9 |
| Cause 11: | Ill defined | 29069 | 3.7 |
| Cause 12: | External | 19597 | 2.5 |
| Cause 13: | Dementia | 52229 | 6.6 |
| Total |  | **794106** | **100.0** |

## Figure A1. Distribution of causes of death

## Table A7. Life Years Lost (LYL; ages 65–99) among women with dementia relative to the general population, stratified by cause of death

| Cause of death | Women – Short-term  education (95% CI) | Women – Long-term education (95% CI) | Women – Intermediate-term  education (95% CI) |
| --- | --- | --- | --- |
| Other causes | 0.04 (–0.01, 0.08) | –0.34 (–0.48, –0.19) | –0.17 (–0.26, –0.09) |
| Cancer | 0.64 (0.60, 0.69) | 0.72 (0.60, 0.83) | 0.76 (0.69, 0.84) |
| Diabetes | –0.11 (–0.14, –0.08) | –0.07 (–0.13, –0.01) | –0.10 (–0.14, –0.06) |
| Mental disorders | –0.06 (–0.07, –0.04) | –0.07 (–0.10, –0.03) | –0.06 (–0.08, –0.03) |
| Nervous system diseases | –0.25 (–0.27, –0.22) | –0.61 (–0.71, –0.52) | –0.39 (–0.44, –0.34) |
| Heart diseases | 0.08 (0.03, 0.13) | 0.09 (–0.01, 0.20) | 0.12 (0.05, 0.18) |
| Cerebrovascular diseases | –0.50 (–0.55, –0.45) | –0.39 (–0.49, –0.28) | –0.44 (–0.51, –0.37) |
| Arterial diseases | 0.00 (–0.02, 0.02) | –0.03 (–0.07, 0.02) | 0.00 (–0.03, 0.03) |
| Respiratory diseases | –0.12 (–0.17, –0.07) | –0.10 (–0.21, 0.01) | –0.09 (–0.15, –0.02) |
| Liver diseases | –0.02 (–0.05, 0.00) | –0.05 (–0.14, 0.02) | –0.06 (–0.09, –0.02) |
| Ill defined | –0.07 (–0.09, –0.04) | –0.13 (–0.20, –0.07) | –0.09 (–0.13, –0.05) |
| External causes | –0.09 (–0.11, –0.07) | –0.05 (–0.11, 0.00) | –0.08 (–0.12, –0.05) |
| Dementia | –3.54 (–3.61, –3.48) | –4.26 (–4.44, –4.07) | –4.24 (–4.36, –4.12) |
| Total Life Years Lost | **–3.99 (–4.03, –3.96)** | **–5.29 (–5.38, –5.20)** | **–4.84 (–4.88, –4.78)** |

The 95% confidence intervals are based on 1000 bootstrap iterations. CI: Confidence interval.

## Table A8. Life Years Lost (LYL; ages 65–99) among men with dementia relative to the general population, stratified by cause of death

| Cause of death | Men – Short-term  education (95% CI) | Men – Long-term education (95% CI) | Men – Intermediate-term  education (95% CI) |
| --- | --- | --- | --- |
| Other causes | –0.02 (–0.10, 0.05) | –0.23 (–0.37, –0.08) | –0.03 (–0.11, 0.05) |
| Cancer | 1.21 (1.13, 1.29) | 1.13 (1.00, 1.25) | 1.33 (1.25, 1.41) |
| Diabetes | –0.16 (–0.20, –0.11) | –0.10 (–0.17, –0.04) | –0.19 (–0.24, –0.15) |
| Mental disorders | –0.12 (–0.15, –0.09) | –0.08 (–0.12, –0.05) | –0.12 (–0.14, –0.09) |
| Nervous system diseases | –0.59 (–0.64, –0.53) | –1.02 (–1.12, –0.89) | –0.85 (–0.91, –0.79) |
| Heart diseases | 0.20 (0.12, 0.28) | 0.19 (0.07, 0.31) | 0.29 (0.21, 0.37) |
| Cerebrovascular diseases | –0.74 (–0.81, –0.66) | –0.60 (–0.71, –0.48) | –0.66 (–0.74, –0.59) |
| Arterial diseases | 0.02 (–0.01, 0.05) | 0.00 (–0.05, 0.05) | 0.01 (–0.02, 0.05) |
| Respiratory diseases | –0.22 (–0.31, –0.15) | –0.26 (–0.37, –0.15) | –0.14 (–0.22, –0.07) |
| Liver diseases | –0.11 (–0.15, –0.07) | –0.14 (–0.21, –0.08) | –0.11 (–0.15, –0.06) |
| Ill defined | 0.01 (–0.03, 0.04) | –0.06 (–0.13, 0.00) | –0.07 (–0.12, –0.03) |
| External causes | –0.07 (–0.10, –0.04) | –0.07 (–0.13, –0.01) | –0.10 (–0.14, –0.07) |
| Dementia | –3.40 (–3.49, –3.30) | –3.77 (–3.94, –3.60) | –3.86 (–3.98, –3.75) |
| Total Life Years Lost | **–3.99 (–4.03, –3.95)** | **–5.01 (–5.09, –4.94)** | **–4.50 (–4.55, –4.46)** |

The 95% confidence intervals are based on 1000 bootstrap iterations. CI: Confidence interval.

## Table A9. Educational differences in life years lost under alternative upper age limits (80 vs. 100 years)

| Sex | Education | LYL, limit 100 years (95% CI) | LYL, limit 80 years (95% CI) |
| --- | --- | --- | --- |
| Women | Short-term | 3.99 (3.96, 4.02) | 1.25 (1.21, 1.28) |
|  | Intermediate-term | 4.84 (4.78, 4.89) | 1.50 (1.45, 1.54) |
|  | Long-term | 5.29 (5.20, 5.38) | 1.55 (1.47, 1.63) |
| Men | Short-term | 3.99 (3.95, 4.03) | 1.63 (1.58, 1.67) |
|  | Intermediate-term | 4.50 (4.46, 4.55) | 1.82 (1.78, 1.86) |
|  | Long-term | 5.01 (4.94, 5.09) | 1.80 (1.74, 1.88) |

Note: 95% confidence intervals are based on 1,000 bootstrap replications. CI: Confidence interval; LYL: Life Years Lost.

## Figure A2. Educational differences in life years lost under alternative upper age limits (80 vs. 100 years)

Tau is the age limit

## Table A10. Mortality Rate Ratios in women and men across education groups, stratified by the underlying age.

| Age group, years | Education | MRR (95% CI) | |
| --- | --- | --- | --- |
|  |  | **Women** | **Men** |
| 65–80 | Short-term | 4.01 (3.93, 4.09) | 3.79 (3.70, 3.88) |
|  | Intermediate-term | 5.13 (4.98, 5.28) | 4.26 (4.16, 4.36) |
|  | Long-term | 6.06 (5.78, 6.36) | 5.29 (5.08, 5.51) |
| 81–90 | Short-term | 2.94 (2.90, 2.99) | 2.87 (2.82, 2.93) |
|  | Intermediate-term | 3.31 (3.23, 3.39) | 3.02 (2.96, 3.09) |
|  | Long-term | 3.59 (3.45, 3.73) | 3.44 (3.32, 3.56) |
| 91+ | Short-term | 2.11 (2.05, 2.16) | 2.17 (2.06, 2.28) |
|  | Intermediate-term | 2.28 (2.19, 2.39) | 2.05 (1.94, 2.17) |
|  | Long-term | 2.25 (2.09, 2.42) | 2.16 (1.98, 2.34) |

Note: MRRs are estimated within strata of education defined at start of follow‑up. The age groups shown in the table reflect the person‑time individuals contribute while they are within each age range. Age is grouped in 2‑year bands starting at 65 years, which means that ages 80 years and 90 years are included as band boundaries. CI: Confidence interval; MRR: Mortality Rate Ratio.
